# How does food addiction influence dietary intake profile?

**DOI:** 10.1371/journal.pone.0195541

**Published:** 2018-04-20

**Authors:** Aylin Ayaz, Reyhan Nergiz-Unal, Damla Dedebayraktar, Asli Akyol, A. Gulden Pekcan, Halit Tanju Besler, Zehra Buyuktuncer

**Affiliations:** Department of Nutrition and Dietetics, Faculty of Health Sciences, Hacettepe University, Ankara, Turkey; University of Kansas Medical Center, UNITED STATES

## Abstract

This study aimed to investigate whether there was any difference in eating pattern, abnormal eating behaviour, obesity and the number of food addiction symptoms according to food addiction presence. A total sample of 851 healthy subjects living in Ankara (n = 360 male, n = 491 female) aged 19–65 years were included in this cross-sectional survey. Data on demographic information, 24-hour dietary recalls, Yale Food Addiction Scale (YFAS), Eating Attitudes Test-26 (EAT-26), and anthropometric measurements were collected through face-to-face interviews. Overall, 11.4% of participants were identified as “food addicted” (F: 13.0%; M: 9.2%). Subjects meeting criteria for ‘food addiction' had higher body mass index (35.1% were obese and 3.1% were underweight), compared to subjects without food addiction (13.1% were obese and 10.2% were underweight) (p<0.05). Abnormal eating attitudes estimated with EAT-26 were determined as 45.5% in males, 37.5% in females and 40.2% in total, among subjects with food addiction. Daily energy, protein and fat intakes were significantly higher in food addicted females, compared to non-addicted females (p<0.05). Participants with food addiction reported significantly more problems with foods, which contain high amounts of fat and sugar, compared to the participants without food addiction. Food addiction behaviour should be considered as a part of efforts towards reducing food related problems involving obesity.

## Introduction

By reaching epidemic proportions globally, obesity has become a major public health problem. More than 1.9 billion adults aged 18 years and over were overweight in 2014, among which over 600 million were obese [1]. According to Turkey Nutrition and Health Survey (TNHS) 2010 data, obesity and overweight prevalence among Turkish adults were 30.3% and 34.6%, respectively [2]. Not only genetics and psychological but also demographical and importantly environmental factors contribute to the increased obesity rates [3]. In this context obesogenic environment is characterized with palatable, energy-dense and inexpensive foods that are available abundantly, and consumption of these foods might be irresistible [4].

With the rapid increase in obesity prevalence, current addiction to food has become a popular topic [5,6]. It has been proposed that food addiction and drug addiction may be similar in terms of cravings, disinhibiting and tolerance [5]. The possible approach of assessing food addiction was based upon the criteria of substance addiction defined by the Diagnostic and Statistical Manual of Mental Disorders-4 (DSM-IV) [4,7]. Experimental studies have found that consumption of both fat and sugar lead to dopamine-related neural activation, producing good mood effect [8–10]. Repeated overconsumption of palatable foods may produce long term neuro-adaptations in brain reward and stress pathways that ultimately promote depressive or anxious responses when those foods are no longer available or consumed [11].

Certain foods may trigger overeating by creating an addiction-like effect on susceptible individuals [12,13]. Foods that are reported to cause addiction are mainly fats, sugar, combination of fat/sugar, or processed foods that are high in salt [13–15]. Energy dense foods, including sugary drinks like beverages, cakes, biscuits, and various salty and savoury snacks are the foods that are most typically associated with reports of food craving and food addiction [12–15].

Addictive potential of certain highly processed foods is considered as a possible factor for some cases of obesity and eating disorders [13,16]. Hence, the most common symptoms of food addiction are loss of control on eating, ongoing consumption in spite of negative outcomes, and incapability of decreasing food intake [17]. Thus, despite food addiction, eating addiction might also play role on binge eating and result in obesity [17–19].

Since there is a lack of knowledge about the addiction to food or eating in human beings, this study aimed to investigate whether any difference in eating pattern, abnormal eating behaviour, body mass index, abdominal obesity, nutrient intake and the number of food addiction symptoms according to the presence of food addiction.

## Materials and methods

### Subjects

A sample of 980 healthy subjects living in Ankara (n = 424 male, n = 556 female) aged 19–65 years were included in this cross-sectional survey. Participants were recruited via emailing and announcements posted in several locations such as cafeteria, sport hall, library and classrooms in Hacettepe University, Ankara. An appointment was provided for each volunteer at Nutrition Education and Research Unit in Department of Nutrition and Dietetics. Researchers provided verbal and visual information on how to respond to items in each questionnaire. Participants who agreed to voluntarily contribute to this study were asked to sign a written consent form in accordance with the Helsinki Declaration. Ethical approval of the study was obtained from the Ethics Board of Hacettepe University, Ankara, Turkey (IRB: GO 14/122).

Students or employees at Departments of Nutrition and Dietetics were excluded from the study, considering that their awareness on eating behaviour might be different than the general population. Pregnant and lactating women were also excluded from the study population as eating behaviours might change at that period of life.

Cut-offs for under and over-reporting of dietary energy intakes via 24-hour dietary recall were calculated using the Goldberg approach as explained by Black [20] and 129 participants were assessed as mis-reporters. As a result, a total of 851 participants (n = 360 male, n = 491 female) with plausible dietary energy intakes were included in the study.

### Measures

Data on demographic information, nutritional habits, 24-h dietary recalls, Yale Food Addiction Scale (YFAS), Eating Attitudes Test-26 (EAT-26), and anthropometric measurements were collected through face-to-face interviews by intern dietitians under the guidance of professional healthcare providers.

#### 24-hour dietary recall

Dietary intake was assessed via 24-hour dietary recall since it has been reported to capture dietary intake with less bias than food-frequency questionnaires [21]. Recalls were performed by trained intern dietitians via face-to-face interviews. A photographic atlas was used to record the type and portion size of the food/fluid/meal [22]. The dietary data was analyzed using BeBIS-6.1 (Nutrition Information Systems Software) software program and total intake of energy, macro and micro nutrients were calculated.

To exclude under-reporters and over-reporters, The Goldberg cut-off principles [23], which have been revised by Black [20], were applied. The Goldberg approach has been widely used to identify invalid reports of energy intake (EI) [23] and it compares total energy expenditure (TEE) expressed as a multiple of basal metabolic rate (BMR) with EI. The TEE/BMR ratio is also known as PAL. In this study BMR was calculated using the Schofield equations for adults based on age, gender, height and weight [24]. Confidence limits (CL) of agreement were applied, based on variation in EI, BMR and PAL, to identify individuals with intakes that are unlikely to represent valid data [20]. Since PAL values were not available, a minimum plausible value of 1.55 was assigned to all individuals, proposed by FAO/WHO/UNU [25] based on the assumption that subjects had a low activity level (i.e., normally active but sedentary).

### Yale Food Addiction Scale (YFAS)

The YFAS was developed to assess food addiction by modifying the seven symptoms of substance dependence using the DSM-IV diagnostic criteria (The Diagnostic and Statistical Manual of Mental Disorders) [26]. The seven symptoms assessed in the scale were: substance taken in larger amounts and for longer period than intended; repeated unsuccessful attempts to quit; too much time spent on eating and food; giving up social, occupational or recreational activities to eat; continuous use despite knowledge of adverse consequences to eating behaviours; tolerance to food; and withdrawal from not eating. A total score was calculated for meeting the criteria for these seven symptoms. Meeting the criteria for three or more of these domains was qualified as “food addiction”. Yale food addiction scale also asks if a person is having problems with certain types of food. This section of the scale contains 26 food items (ice cream, chocolate, apples, doughnuts, broccoli, cookies, cake, candy, white bread, rolls, lettuce, pasta, strawberries, rice, crackers, chips, pretzels, french fries, carrots, steak, bananas, bacon, hamburgers, cheeseburgers, pizza and soda pop) [26]. In the current study, the Turkish version of YFAS, which was validated by Buyuktuncer et al., was used [27].

### Eating Attitudes Test-26 (EAT-26)

Eating Attitudes Test-26 (EAT-26), widely used to measure symptoms of disordered eating both of the anorexic and bulimic variants, was developed by Garner et al. [28]. It includes 26 items in which the frequencies of attitudes and beliefs are rated by using a 6-point scale. Participants with scores of 20 or greater are considered at high risk for meeting the criteria for an eating disorder. In the current study, the Turkish version of EAT-26, which was validated by Baş et al., was used [29]. The EAT-26 is based on an original Eating Attitudes Test (EAT-40), which was validated in Turkish by Savaşır & Erol in 1989 [30].

### Anthropometric measurements

The body weights of the participants, wearing minimal clothing without shoes, were measured with a portable scale. Height was measured with a wall-mounted stadiometer. Waist circumference was measured from the lowest circumference in the middle of the iliac prominence and the lowest rib of the individual with inflexible measuring tape. Hip circumference was measured parallel to the ground from the highest point on the hip with inflexible measuring tape. All measurements were obtained as previously described by trained intern dietitians [31]. Body Mass Index (BMI: weight/height^2^, kg/m^2^) and waist/hip ratio were calculated and evaluated according to the classification of the World Health Organization [32,33].

### Statistical analysis

Results were expressed as the median, min and max. Significance of differences was determined with Mann–Whitney U test or chi-square (*X*^*2*^) test as required. For the correlation analysis, the Spearman Correlation Test was performed. Statistical significance was set at p<0.05 and data analysis was performed with the Statistical Package for Social Sciences software (SPSS version 23.0, Chicago, USA).

## Results

General characteristics of the participants categorized by presence of food addiction were presented in Table 1. Overall, 11.4% of the participants were identified as “food addicted” (F: 13.0%; M: 9.2%) (Data is not shown). Since the significance has the same tendency in both genders, the p values for the total participants were shown in Table 1. General characteristics such as age, occupation, marital and physical activity status were not different significantly, except for educational status. Among the participants who studied at least university education, the prevalence with food addiction was 24.7%, whereas it was 31.7% among those without food addiction. As education level increased, food addiction decreased significantly. Addiction-like behaviours such as smoking and alcohol consumption were not significant either (p>0.05). Mean age of the participants with and without food addiction were 34.6±12.8 and 31.8±12.2 years among males (p>0.05); and 29.9±10.2 and 31.1±12.1 years among females, respectively (p>0.05) (Data is not shown).

**Table 1:** General characteristics of the participants categorized by presence of food addiction by gender.

Variable, n (%)^a^		FAD			NFA			χ ^2^*/Z factor*	*P value*
		Male (n = 33)	Female (n = 64)	Total (n = 97)	Male (n = 327)	Female (n = 427)	Total (n = 754)		
Age (years)	19–39	22 (66.7)	49 (76.6)	71(73.2)	243 (74.3)	320 (74.9)	563 (74.7)	0.098	0.754
	40–65	11 (33.3)	15 (23,4)	26 (26.8)	84 (25.7)	107 (25,1)	191 (25.3)		
Marital Status	Married	17 (51.5)	25 (39.1)	42 (43.3)	123 (37.6)	158 (37.0)	281 (37.3)	2.941	0.230
	Single	15 (45.5)	36 (56.3)	51 (52.6)	198 (60.6)	258 (60.4)	456 (60.5)		
	Divorced	1 (3.0)	3 (4.7)	4 (4.1)	6 (1.8)	11 (2.6)	17 (2.3)		
Education	No schooling to primary school	4 (12.1)	12 (18.7)	16 (16.5)	7(2.1)	44 (10.3)	51 (6.8)	12.946	0.017*
	Secondary school	2 (6.1)	1 (1.6)	3 (3.1)	11 (3.4)	18 (4.3)	29 (3.8)		
	High school	16 (48.5)	38 (59.4)	54 (55.7)	180 (55.0)	255 (59.7)	435 (57.7)		
	University/postgraduate	11 (33.3)	13 (20.3)	24 (24.7)	129 (39.4)	110 (25.8)	239 (31.7)		
Occupation	No work, staying at home	-	15 (23.4)	15 (15.5)	-	96 (22.5)	96 (12.7)	3.873	0.794
	Student	6 (18.2)	29 (45.3)	35 (36.1)	109 (33.3)	193 (45.2)	302 (40.1)		
	Government employee (officer)	6 (18.2)	12 (18.8)	18 (18.6)	73 (22.3)	57 (13.3)	130 (17.2)		
	Labourer	3 (9.1)	1 (1.6)	4 (4.1)	24 (7.3)	14 (3.3)	38 (5.0)		
	Retired	5 (15.2)	-	5 (5.2)	31 (9.5)	11 (2.6)	42 (5.6)		
	Self-employment	8 (24.2)	2 (3.1)	10 (10.3)	33 (10.1)	16 (3.7)	49 (6.5)		
	Other	5 (15.1)	5 (7.9)	10 (10.4)	57 (17.5)	40 (9.3)	97 (12.9)		
Smoking Status	Non-smoker	19 (57.6)	44 (68.8)	63 (64.9)	187 (57.2)	346 (81.0)	533 (70.7)	4.104	0.129
	Ex-smoker	4 (12.1)	3 (4.7)	7 (7.2)	49 (15.0)	26 (6.1)	75 (9.9)		
	Smoker	10 (30.3)	17 (26.6)	27 (27.8)	91 (27.8)	55 (12.9)	146 (19.4)		
Alcohol consumption		11 (34.4)	10 (30.3)	12 (18.8)	90 (27.4)	91 (27.8)	55 (12.9)	0.597	0.440
Physical Activity	Regular exercisers	10 (30.3)	15 (23.4)	25 (25.8)	95 (29.1)	92 (21.5)	187 (24.8)	0.043	0.835
	Exercise (minutes/day)^b^	45.0 (30.0–120.0)	30.0 (19.0–60.0)	45.0 (19.0–120.0)	45.0 (10.0–130.0)	30.0 (19.0–60.0)	45.0 (10.0–130.0)	-0.610	0.545

^a^ Data are presented as count(%).

^b^ Data are presented as Median (Min-Max).

FAD = ‘Food addicted’ (≥3 symptoms + satisfying clinical impairment/distress criteria), NFA = non-addicted. Significance was calculated for all variables (age, marital status, education, occupation, smoking status, alcohol consumption and regular exercisers) for comparison between FAD and NFA individuals (total) with chi-square (X^2^) test for nominal data analysis.

* = ***p*** < 0.05.

Abnormal eating attitudes (EAT-26 SCORE ≥20) estimated with EAT-26 were determined 45.5% in males, 37.5% in females and 40.2% in total of the participants with food addiction and 10.1% in males, 12.2% in females and 11.3% in total without food addiction (p = 0.000) (Data is not shown). Anthropometric measurements categorized by presence of food addiction were presented in Table 2. Waist and hip circumferences were higher in food addicted participants compared to the ones without food addiction in both genders, and waist/hip ratio was significantly higher in food addicted females. As an abdominal obesity indicator median waist circumferences were 96cm in males and 89.5 cm in females with food addiction while they were 90cm in males and 78cm in females without food addiction (p<0.05). Body weight categorised according to BMI showed that 35.1% were obese and 3.1% were underweight with food addiction whereas 13.1% were obese and 10.2% were underweight without food addiction (p<0.05) (Table 2).

**Table 2:** Anthropometric measurements categorized by presence of food addiction by gender.

	FAD				NFA			
Measurements	Male (n = 33)	Female (n = 64)	Total (n = 97)	Male (n = 327)	Female (n = 427)	Total (n = 754)	χ ^2^*/Z factor*	*P value*
Waist (cm) ^a^	96.0 (67.0–123)	89.50 (58.0–125.0)	92.0 (58.0–125.0)	90.0 (59.0–123.0)	78.0 (52.0–131.0)	84.0 (52.0–131.0)	M:-2.393	0.017*
							F:-3.509	0.000*
Hip (cm) ^a^	108.0 (85.0–120.0)	107.5 (82.0–135.0)	108.0 (82.0–135.0)	101.0 (60.0–135.0)	99.0 (60.0–147.0)	100.0 (60.0–147.0)	M:-2.488	0.013*
							F:-3.480	0.001*
Waist/Hip Ratio ^a^	0.90 (0.74–1.14)	0.84 (0.59–1.07)	0.86 (0.59–1.14)	0.90 (0.60–1.15)	0.79 (0.56–1.20)	0.84 (0.56–1.20)	M:-0.754	0.451
							F:-2.062	0.039*
BMI (kg/m^2^) ^a^	27.2 (17.4–36.9)	26.7 (17.0–47.6)	26.8 (17.0–47.6)	25.4 (16.4–38.9)	22.6 (15.4–43.8)	23.7 (15.4–43.8)	M:-3.040	0.002*
							F:-4.277	0.000*
Weight Categories n (%) ^b^^,^^c^								
Underweight (n = 90)	1 (3.0)	2 (3.1)	3 (3.1)	20 (6.1)	57 (13.3)	77 (10.2)	M:13.609	0.002*
Normal—weight (n = 457)	8 (24.2)	26 (40.6)	34 (35.1)	138 (42.2)	230 (53.9)	368 (48.8)		
Pre—obese (n = 282)	12 (36.4)	14 (21.9)	26 (26.8)	133 (40.7)	77 (18.0)	210 (27.9)	F:19.533	0.000*
Obese (n = 151)	12 (36.4)	22 (34.4)	34 (35.1)	36 (11.0)	63 (14.8)	99 (13.1)		

^a^ Significance was calculated with Mann Whitney U test for ordinal data analysis by gender expressed median (min-max).

^b.^ Chi-square (χ2) test for nominal data analysis by gender expressed as n (%).

^c^ BMI (kg/m^2^): <18.5, underweight; 18.5–24.99 normal–weight; 25.0–29.99 pre-obese; ≥30 obese (classification according to WHO.

FAD = ‘Food addicted’ (≥3 symptoms + satisfying clinical impairment/distress criteria), NFA = non-addicted.

* = ***p*** < 0.0.

**Fig 1:**
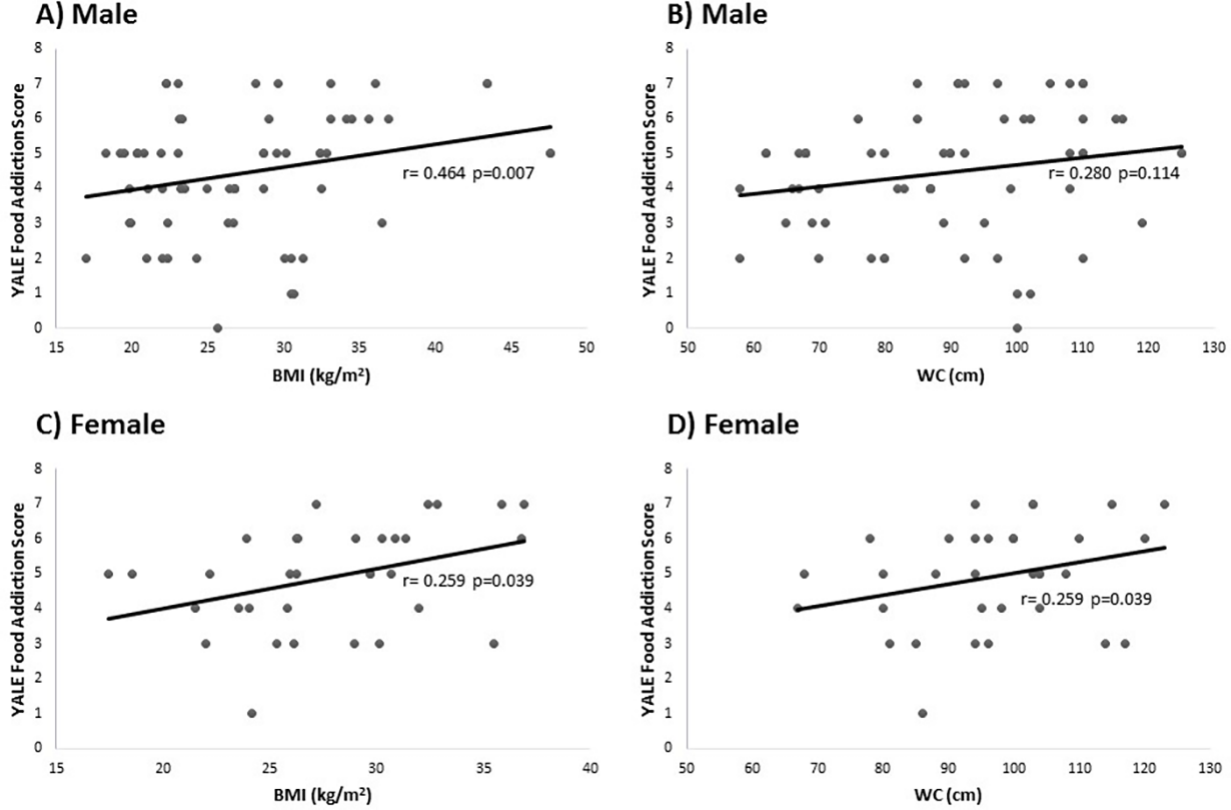
The correlations between YALE food addiction score and body mass index (BMI) or waist circumference (WC) of the participants with food addiction based on the gender. Scatter plot of food addiction scores and (A) BMI (kg/m^2^) (r = 0.464, p = 0.007) and (B) WC (cm) (r = 0.280, p = 0.114) among male participants with food addiction (n = 33). Scatter plot of food addiction scores and (C) BMI (kg/m2) (r = 0.259, p = 0.039) and (D) WC (cm) (r = 0.259, p = 0.039) among female participants with food addiction (n = 64).

Correlations between YALE food addiction scores and body mass indexes or waist circumferences of the participants with food addiction based on the gender was presented in Fig 1. The scatter plot graphs showed that food addiction scores were positively correlated with BMI (r = 0.464, p = 0.007), but were not correlated with WC (r = 0.280, p = 0.114) among males. On the other hand, among females, food addiction scores were correlated with both BMI (r = 0.259, p = 0.039) and WC (r = 0.259, p = 0.039).

Daily dietary nutrient intakes of the participants categorized by presence of food addiction were shown in Table 3. Daily energy intake and the contributing macronutrients such as protein and fat intakes were significantly higher in food addicted females compared to the non-addicted females (p<0.05). In parallel, fatty acid profile and cholesterol amount were significantly higher in food addicted females (p<0.05). The intake of micronutrients (vitamins and minerals) such as; vitamin A, vitamin E, vitamin B_12_, magnesium, iron and zinc were significantly higher in females with food addiction (p<0.05) (Table 3).

**Table 3:** Daily dietary energy and nutrient intakes of participants categorized by presence of food addiction.

	Male (n = 360)			Female (n = 491)		
Energy and Nutrients^a^	FAD (n = 33)	NFA (n = 327)	*P value*	FAD (n = 64)	NFA (n = 427)	*P value*
Energy (kcal)	2499.7 (1345.0–4343.8)	2587.8 (1426.3–4692.0)	0.719	2377.2 (1213.9–4556.5)	2071.1 (1121.3–4118.9)	0.006*
Protein (g)	93.4 (43.2–223.9)	95.6 (32.8–267.6)	0.313	82.1 (51.1–144.1)	73.4 (28.3–160.8)	0.009*
Fat (g)	102.6 (64.2–220.2)	109.1 (43.1–261.6)	0.838	102.9 (51.2–208.3)	87.0 (33.5–260.4)	0.000*
Saturated fats (g)	32.2 (17.6–66.7)	32.9 (10.4–193.5)	0.833	29.5 (15.2–65.7)	25.6 (9.0–56.7)	0.002*
MUFA (g)	33.0 (18.1–72.5)	33.5 (13.2–243.4)	0.890	29.5 (14.4–76.6)	26.3 (10.3–66.9)	0.002*
PUFA (g)	37.6 (15.9–79.4)	35.3 (7.9–261.9)	0.993	35.3 (12.9–73.9)	29.3 (6.3–150.0)	0.000*
Cholesterol (mg)	333.7 (131.8–781.5)	284.7 (11.0–1338.5)	0.071	278.6 (33.1–773.5)	213.7 (14.7–914.1)	0.001*
Carbohydrate (g)	293.8 (95.6–525.0)	295.1 (98.8–620.0)	0.548	243.6 (100.7–575.0)	235.6 (81.7–651.9)	0.170
Sucrose (g)	34.6 (2.5–11.1)	44.9 (2.1–245.2)	0.067	41.7(6.6–155.0)	37.7 (4.0–208.4)	0.242
Fibre (g)	29.8 (6.6–62.4)	29.6 (7.2–82.1)	0.662	29.8 (10.9–86.6)	25.5 (8.5–96.4)	0.016*
Vitamin A (μ g)	1989.4 (539.9–7591.4)	1968.7 (135.8–9779.9)	0.892	2090.1 (754.4–6364.9)	1893.5 (196.6–8248.6)	0.046*
Vitamin E (mg)	28.1 (8.9–73.2)	28.3 (5.5–122.6)	0.903	29.5 (10.9–71.5)	25.9 (5.1–152.1)	0.003*
Thiamine (mg)	1.32 (0.39–2.63)	1.34 (0.56–3.96)	0.711	1.28 (0.54–2.40)	1.12 (0.47–3.00)	0.059
Riboflavin (mg)	1.90 (0.80–5.55)	1.96 (0.72–5.13)	0.753	1.81 (1.07–3.95)	1.60 (0.61–9.04)	0.100
Folic acid (μg)	423.7 (123.2–770.5)	397.8 (22.8–1028.7)	0.703	372.7 (33.9–701.0)	333.0 (32.6–900.9)	0.067
Vitamin B_12_ (μg)	6.2 (1.9–59.9)	5.4 (0.5–74.6)	0.312	4.4 (0.8–38.0)	3.6 (0.2–40.6)	0.006*
Ascorbic acid (mg)	113.0 (48.8–262.4)	107.5 (1.4–529.6)	0.657	117.1 (2.9–323.3)	110.5 (1.8–379.6)	0.312
Potassium (mg)	3085.8 (1222.4–5938.5)	3027.6 (1254.9–8263.9)	0.570	3027.2 (1288.4–6112.9)	2702.4 (836.4–6216.3)	0.082
Calcium (mg)	800.6 (297.6–1440.8)	848.8 (211.3–3889.2)	0.580	729.7 (445.0–2097.0)	727.0 (219.2–3909.2)	0.475
Magnesium (mg)	385.1 (120.1–887.4)	399.8 (141.9–1434.6)	0.769	381.6 (159.0–1282.9)	339.9 (94.0–1398.1)	0.048*
Iron (mg)	18.1 (5.7–37.4)	17.5 (7.6–45.1)	0.549	17.4 (8.2–34.9)	15.2 (4.3–39.4)	0.009*
Zinc (mg)	12.6 (4.1–27.3)	12.9 (4.9–53.0)	0.335	11.6 (6.7–31.7)	10.0 (3.9–25.4)	0.008*

^a^ Significance (P < 0.05) was calculated with Mann-Whitney U test for ordinal data analysis by gender expressed median (min-max).

FAD = ‘Food addicted’ (≥3 symptoms + satisfying clinical impairment/distress criteria), NFA = non-addicted. MUFA: Monounsaturated fatty acids, PUFA: Polyunsaturated fatty acids.

* = ***p*** < 0.05.

Correlations between YALE food addiction scores and body mass index or waist circumferences of the participants with food addiction based on the gender was presented in Fig 1. The scatter plot graphs show that food addiction scores and BMI (r = 0.259, p = 0.039) and WC (r = 0.259, p = 0.039) among females were positively correlated. Food addiction scores and BMI (r = 0.464, p = 0.007) were positively correlated among males with food addiction.

**Fig 2:**
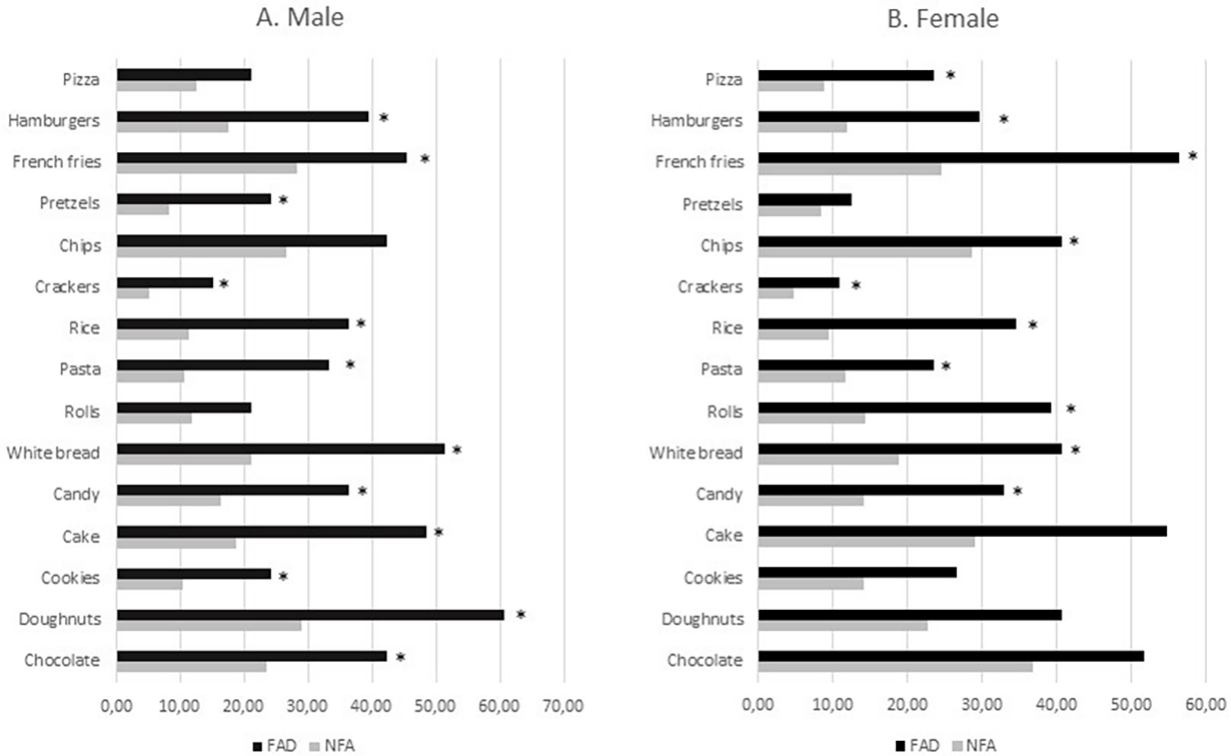
Percentages of participants who reported having problems with certain types of food items in YFAS according to presence of food addiction. Chi-square (χ2) test was performed for nominal data analysis by gender presented as (%). FAD = ‘Food addicted’ (≥3 symptoms + satisfying clinical impairment/distress criteria), NFA = non-addicted.* = *p* < 0.05.

Percentages of participants who have problems with certain types of food items in Yale food addiction scale, based on whether they had food addiction or not, were presented in Fig 2. Males with food addiction reported having significantly more problems with; chocolate, doughnuts, cookies, cake, candy, white bread, pasta, rice, crackers, pretzels, french fries and hamburgers compared to males without food addiction (p<0.05). Females with food addiction reported to have significantly more problems with; chocolate, doughnuts, cookies, cake, candy, white bread, rolls, pasta, rice, crackers, chips, french fries, hamburgers and pizza compared to females without food addiction (p<0.05).

## Discussion

Although evidence of food addiction continues to grow, limited number of studies have examined whether food addiction or addictive-like eating is attributed to obesity related eating behaviours or not [13,34] As a response to the need for a reliable and validated method to define food addiction hypothesis and its contribution to eating behaviour problems, YFAS was developed by Gearhardt et al in 2009 in English [26]. French, German and Danish versions of this scale in general population were used in literature as well [35]. The Turkish version of the YFAS usage in general population was limited with two studies [36,37]. Pursey et al reported that studies are yet to use YFAS in combination with specific foods or nutrients [38]. Current study, which evaluated dietary intake of energy and nutrients according to presence of food addiction, was assessed via YFAS.

The recurrent access to food may be parallel with eating profile of some individuals with restrained or disordered eating behaviour [39]. The identification of a potentially addictive profile of hyper-palatable processed fatty, salty or sweet foods is essential for understanding the food or eating addiction construct and for making nutrition policies for public health [13]. In this study, among all participants, 11.4% were identified as food addicted. Food addiction was more prevalent among female participants (F: 13.0%; M: 9.2%). Overall percentage of food addiction was similar to the findings of Gearhardt et al [26] who reported the percentage of food addiction among general public as 11.6%. Yu and Tan [40] reported prevalence of food addiction as 10.3% among college students which was slightly lower than both our and Gearhardt’s findings. On the other hand, Pursey et. al. [35] reported the weighted mean prevalence of food addiction (FA) as 19.9% for adults across 20 studies.

Research shows that, eating disorder patients seem to be more likely to develop addictive eating patterns [41]. In the current study, according to EAT-26 scale, abnormal eating attitude was higher among those with food addiction compared to the participants without food addiction. Hence, increased addiction to food was thought to be associated with an increase in abnormal eating behaviour. This corresponds with the study which suggests that the presence of “food addiction” may indicate a more severe presentation of eating disorders, associated with factors such as more frequent binge eating episodes, and earlier onset of problematic eating behaviour [8].

Energy dense junk foods may contribute to problematic eating behaviour and potential weight gain in vulnerable individuals [18,42]. It was demonstrated that food addiction contributes to severity of obesity and body composition measurements among normal weight in terms of obese individuals in the general population with higher rate in women as compared to men [43]. Pursey et al reported that (35) food addiction prevalence was also as twice as high in the overweight/obese population, compared to those with a healthy BMI (24.9% and 11.1% respectively). Obregon et al [44] also observed a higher prevalence of food addiction (30%) among those who were classified as obese. In this study, obesity prevalence was higher in food addicted subjects and the existence of obesity was similar in both sexes. Nutritional dependence was associated with higher energy intake among females and increased abdominal adiposity in both sexes. Body mass index, abdominal obesity, waist and hip circumferences were higher in subjects with food addiction compared to the participants with no food addiction in both genders. In the absence of food addiction, the underweight rate was higher (Table 2). BMI and YFAS symptom counts were small-to-moderate positive predictors for this association shown by others as well [13,45]. The results demonstrated that food addiction might contribute to the severity of the obesity, and visceral fat deposition composition measurements shown formerly by others [36, 43, 45]. Prevalence of food addiction and measures of adiposity (body fat, BMI) have been found to be positively correlated [11,35]. In this study BMI was positively correlated with YFAS scores among both male and female participants food addiction, while correlation between WC and YFAS scores were significant only among females (Fig 1).

Not all foods are equally implicated in addictive-like eating behaviour, and highly processed foods, which may share characteristics with drugs of abuse (e.g. high dose, rapid rate of absorption) appear to be particularly associated with food addiction [13]. The food environment has changed dramatically with the incursion of hyper-palatable foods that are produced to exceed the rewarding properties of traditional foods (e.g. vegetables, fruits, nuts) by increasing fat, sugar, salt, flavours and food additives to high levels [39].

Highly processed, hyper-palatable energy–dense foods with combinations of fat and sugar appear most likely to trigger and addictive-like response [13]. Pursey et al reported that higher YFAS food addiction scores were associated with higher percentage energy intake (E%) from energy-dense, nutrient-poor foods [34]. “Junk” foods, such as candy, chocolate, cookies, ice-cream and cakes, were also associated with addictive-like eating in self-reported food addicts [46]. In the current study, participants with food addiction reported having significantly more problems with certain types of food items with high fat and sugar content (Fig 2). In our study, energy intake of food addicted females was significantly higher, supporting previous findings of Gearhardt et al [4]. Few studies have investigated the specific food components that lead to an addictive-like response in humans [38]. Similar to our study, Pedram et al. found that daily intakes of protein and fat were significantly higher in individuals meeting the YFAS criteria for food addiction [43]. Another study conducted with obese individuals reported significantly higher intakes of fat and sugar in those identified as food addicted according to the YFAS [47]. On the other hand, in a sample of 462 young Australians, no relationship between intake of sugars and food addiction was identified [34]. In the current study, sugar consumption was not significantly different between participants with and without food addiction. Addictive-like behaviours occurred to a lesser degree when the diet was supplemented with sugar only than diets with a combination of sugar and fat [14].

In the current study, the intake of certain micronutrients (vitamin A, vitamin E, vitamin B_12_, magnesium, iron, zinc) was significantly higher in females with food addiction. Pursey et al. reported in their study that there were no significant associations between the presence of food addiction and micronutrients in young adults aged 18–35 years [34]. It has been suggested that all individuals displaying addictive-like eating may not be following a specific dietary pattern, and overeating foods with differing composition may not result in single micronutrient excesses or deficiencies [38]. Pedram ad Sun showed that overweight/obese individuals with food addiction consumed more sodium, potassium, calcium, selenium, vitamin D and gamma-tocopherol compared to overweight/obese individuals without food addiction [47].

This study has some strengths and shortcomings to be considered. To our knowledge, this is the first study to evaluate the relation between food addiction and nutritional intake assessed by 24-hour dietary recall. Anthropometrical measurements were not based on subjective statements and were taken by trained intern dietitians with appropriate methods under the supervision of the researchers. First shortcoming of the present study was that, it was conducted in Ankara, so the results cannot be generalized to Turkish population. Secondly, although this study was carried out among adult population, the number of participants over 40 years of age was lower compared to young adults. Having equal numbers reflecting each age group would be useful in future studies. Third, food consumption of female participants during different phases of menstrual cycle were not assessed in our study. Longitudinal studies evaluating food consumption and food addiction symptoms during different phases of menstrual cycle (menstrual, follicular, ovulation, luteal phases) should be planned. Fourth, a single PAL value of 1.55 was used in order to define participants with non-plausible dietary energy intakes according to Goldberg principle. Using an objective method such as a multisensory armband or an accelerometer to assess PAL values would have been more reliable. Lastly, since mental status is an important factor that affect food intake, future studies involving clinical evaluation of emotional parameters in a multidisciplinary approach would help in interpreting food addiction behaviour data.

## Conclusions

Obesity epidemic is a major global health problem. Desire for highly energy-dense and reinforcing foods is reported to be contributing to weight gain by recent studies. Increased consumption of high-fat and high-sugar foods have resulted in increased number of food addicts in developed and developing countries. Consequently, presence of food addiction is related with energy dense food consumption and obesity; mainly abdominal fat accumulation. Since food addiction is predominantly influenced and provoked by compulsive behaviour and emotional and environmental factors such as stress, socio-cultural status and habitual characteristics, these factors should be taken into account in future studies. In addition, future randomised controlled researches would contribute to identifying characteristics, combinations and concentrations of ingredients which are potentially addictive. Efforts to improve nutrition knowledge and promote consciousness about food label reading may assist individuals to make more informed food choices. Corporate responsibility, public health approaches, environmental change and global efforts seem to be essential in reducing food and substance related problems involving obesity and other addiction symptoms.

## Supporting information

S1 File Dietary intake and food addiction data set. (SAV)
